# Phytochemical and proximate content of *Carapa procera* bark and its antimicrobial potential against selected pathogens

**DOI:** 10.1371/journal.pone.0261755

**Published:** 2021-12-23

**Authors:** Derrick Ansah Owusu, Alfred Elikem Kwami Afedzi, Lydia Quansah

**Affiliations:** Department of Biotechnology, Faculty of Biosciences, University for Development Studies, Tamale, Ghana; Bangabandhu Sheikh Mujibur Rahman Agricultural University, BANGLADESH

## Abstract

Medicinal plants represent a great source of antimicrobial and phytochemical constituents which are increasingly used to treat microbial infections and other ailments such as tuberculosis, anemia, and trachoma. Despite the use of antibiotics, antimicrobial resistance continues to be a world issue, in as much as nutrition. This study investigated the presence of phytochemicals, proximate compositions, and antimicrobial activity of methanolic extract of *Carapa procera* bark. The bark of *Carapa procera* was collected, cleaned and air dried for 72 h. The powder obtained was treated with diethyl ether and soaked in methanol (99%) for 72 h to obtain crude extract. The extract was used to test for the presence of phytochemicals and antimicrobial activities. The raw bark was used for proximate analysis. The result showed presence of steroids, tannins and saponins, but no alkaloids present. The 100 mg/mL extract had the highest inhibition zone on all tested organisms from 24.00 ± 0.94 to 26.67 ± 1.18, and 50 mg/mL showed the least (16.67 ± 1.24) on *Candida albicans*. *Staphylococcus aureus* showed the lowest minimum inhibition concentration (MIC) of 3.12 mg/mL, whereas the Gram-negative bacteria exhibited variations in their sensitivity with *E*. *coli* having the highest MIC of 25 mg/mL. The extract had high MIC (6.25 mg/mL) on *Candida albicans* than clotrimazole (50 mg/mL). The proximate compositions of *Carapa procera* were moisture (6.07 ± 0.07%), ash (12.46 ± 0.46%), crude protein (9.54 ± 0.12%), crude fat (1.42 ± 0.06%) and carbohydrate (70.50 ± 0.35%). The energy value was 1413.17 kj. Thus, *Carapa procera* possesses both antimicrobial and nutritional potentials worth exploring and domesticating for sustainable management and conservation.

## Introduction

Medicinal plants are of high value to the health of humans and animals due to their pharmacological potential. Medicinal plants contain phytochemicals such as tannins, saponins, alkaloids, terpenoids and flavonoids which are effective against microbial infections [1], and other biological roles including anti-ulcer, anti-inflammatory and antioxidant activity [2]. Antimicrobial Resistance (AMR) being a global issue, is estimated to be associated with increase mortality rate in Africa and Asia by 2050 [3] due to inappropriate use of antibiotics or antifungals indicating downsizing of effective antibiotics, and therefore, urgent measures are needed [4]. About 80% of the populations of developing countries depend on traditional health care approach which employs the use of plant extracts [5] and serve as a first choice of treatment [5].

In Ghana, the spread of multi drug resistance *Staphylococcus aureus* recorded 34.8% [6]. Traditional mode of treatment in healthcare has compelled researchers to work on the mechanisms of medicinal plants on microbes [7]. There has been increasing research in the role of traditional medicinal plants in the control or prevention of diseases. Nutritional factors such as protein, carbohydrate and minerals are also required to fight diseases, and this add benefit to the application of herbal plants in medicine.

*Carapa procera* (D.C.) is a medicinal woody tree which belongs to the Meliaceae family. It is usually located along gallery forest in savanna regions and humid forest in western and central Africa [8]. *Carapa procera* has being used in herbal medicine, cosmetics, veterinary medicine, and as insecticide [9, 10]. *Carapa procera* is called “crabwood” in English, and “Kwaebese” in the Ghanaian Akan language. In Ghana, the leaf of this plant when dried is effective for treating hypertension, whereas the stem and the bark is used to treat tuberculosis, anemia, syphilis, and trachoma [11]. The leaves and seeds of the plant have been extensively exploited, but little work is done on the bark for their therapeutic and nutritional properties. There is no information on the proximate composition of *C*. *procera* bark, which could add nutritional benefit to its application.

In consonance to the medicinal value of *C*. *procera* and the increasing intake of natural products, the objective of this study seeks to evaluate the proximate composition and phytochemicals in *Carapa procera*, and its antimicrobial activity on some selected pathogens.

## Material and methods

### Collection and preparation of plant material

The barks of *Carapa procera* were collected during the wet season (April to October) in the year 2018 from Akim Oda forest in the Eastern Region of Ghana. The barks were collected by peeling the stem off with machete. The sampled barks were washed with tap water to get rid of any debris and then air dried at a relative humidity (70%) and temperature (30°C) for three weeks before being crushed with mortar and pestle. The duration for the drying was to bring the moisture content below 10% to preserve its quality, inhibits the growth of microorganisms and chemical alterations during dried storage [12]. The crushed sample was sieved (5 mm sieve) to obtain powder which was then stored in air-tight container at room temperature (29°C) and relative humidity (72%) until used. The powder (150 g) was mixed with 150 mL of diethyl ether and allowed to stand at room temperature for 24 h to remove any fat. The residual was dried in an oven (J. P Selecta, Abrera, Spain) at 42°C for 24 h after filtration with Whatman No. 1 filter paper and the liquid lipid fraction discarded.

### Methanolic extraction

The methanolic extraction was done using 50 g of the fat free powder which was soaked in 150 mL of methanol (99%) at room temperature for 72 h and filtered. The filtrate was used for qualitative test for the presence of phytochemicals.

For antimicrobial assay, the filtrate was further evaporated using a vaporizer (Gallenkamp Oven 300 Plus Series, Germany) at 40°C for 48 h and powder extract was obtained. Methanol concentrations of the powder were prepared for zone of inhibition and minimum inhibition concentration (MIC).

### Phytochemical analysis

To test for alkaloids Mayer’s test was performed. Mayer’s reagent (2 mL) was added in drops to 2 mL of the extract in test tube and then shaken. Formation of greenish or creamy color precipitation indicates the presence of alkaloids [13, 14].

Sodium hydroxide test for flavonoids was performed by adding 2 mL of 10% NaOH solution to 2 mL of the extract in test tube. The formation of yellow or orange color indicates the presence of flavonoids [15].

Frothing test was used to analyze saponins by mixing 2 mL of the extract with 20 mL of distilled water in a test tube and shaken vigorously for 15 min. It was allowed to stand for 30 min and the appearance of foam on top indicates the presence of saponins [16].

The presence of steroids was determined using the Liebermann-Burchard’s test. The extract (2 mL) was mixed with 2 mL of chloroform. The solution was mixed with 2 mL of acetic anhydride and 2 mL of concentrated sulphuric acid from the side of the test tube. The appearance of dark green coloration indicates the presence of steroids [17].

Salkowski test for terpenoids was done by mixing 2 mL of the extract in 2 mL of chloroform. Concentrated sulphuric acid (3 mL) was carefully added. Formation of reddish-brown color indicates the presence of terpenoids [18].

The ferric chloride test was used to test for tannins by boiling 2 mL of the extract in test tube for 5 min and allowed to cool. Drops of 1 mL of 5% ferric chloride was added and the appearance of green-blackish color indicates the presence of tannins [19].

Baljet test was performed for the presence of glycosides by adding 2 mL of sodium picrate solution to 2 mL of the extract. The appearance of yellow to orange color shows the presence of glycosides [20].

### Antimicrobial activity

#### Organisms

The test organisms used were *Escherichia coli* (ATCC 25922), *Pseudomonas aeruginosa* (ATCC 4853), *Streptococcus pyrogenes*, *Staphylococcus aureus* (ATCC 25923), and *Candida albicans*. The pure cultures were pre-enriched in nutrient broth at 37°C for 24 h and used as inoculum.

#### Antimicrobial assay

Methanol concentrations of extract used were 100 mg/mL, 50 mg/mL, 25 mg/mL, and 12.5 mg/mL. The agar well diffusion method was employed for antimicrobial assay using 1 mL of each inoculum spread on nutrient agar plates. A sterile 12 mm diameter cork borer was used to create wells in the inoculated plates, and each well filled with 100 *μ*L of the various extract concentrations. The plates were allowed for proper diffusion in the wells for 30 min and then incubated at 37°C for 24 h. Ciprofloxacin and clotrimazole were used as positive control for the bacteria and the fungi isolates, respectively. These were triplicated. After incubation, the zones of inhibition (clear zones around the wells) were measured using a meter rule.

#### Minimum Inhibition Concentration (MIC)

The concentrations of the extract used were 50 mg/mL, 25 mg/mL, 12.5 mg/mL, 6.25 mg/mL, 3.12 mg/mL, 1.56 mg/mL, 0.78 mg/mL, and 0.39 mg/mL, with ciprofloxacin and clotrimazole as positive control. Here, 100 *μ*L of double strength nutrient broth (DSNB) was pipetted into 96 mm wells of two microtiter plates and incubated for 24 h at 37°C. Then, 50 *μ*L of 3-(4,5-dimethylthiazol-2-yl)-2,5-diphenyl-2H-tetrazolium bromide (MTT) dye was loaded into the wells and incubated for another 30 min and color changed observed. The materials used for the MIC are shown (Table 1).

**Table 1:** Minimum Inhibition Concentration (MIC) test materials.

Materials	Tests							
^a^DSNB (*μ*L)	100	100	100	100	100	100	100	100
Extract (*μ*L)	121	61	30	15	8	62.5	31	16
Water (*μ*L)	4	64	95	110	117	62.5	94	109
Organism (*μ*L)	25	25	25	25	25	25	25	25
Volume (*μ*L)	250	250	250	250	250	250	250	250
Concentration (mg/mL)	50	25	12.5	6.25	3.13	1.56	0.78	0.39

^a^Double Strength Nutrient Broth.

### Proximate analyses

The moisture, crude protein, crude fat, and ash of bark powder of *Carapa procera* were determined by the standard methods of the Association of Official Analytical Chemists [21]. The moisture content was determined by weighing 3.5 g of the sample into a moisture can and dried in hot air oven at 105°C for 3 h 30 min until a constant weight was obtained. Amount of ash was determined by ashing 3.5 g of the sample at 550°C for 4 h. The protein content was determined using Kjeldahl method, where the nitrogen value (N) was multiplied by the conversion factor of 6.25. The Soxhlet extraction method was used to determine crude fat. The proximate was done in triplicates with values presented in percentage. Carbohydrate content (CHO%) was determined using subtraction method by taking the difference of the sum of all the proximate composition from 100 (CHO% = [100 − moisture%–protein%–fat%–ash%]) [22, 23].

### Energy value

The Atwater general factor system (17 kj/g (4.0 kcal/g) for protein, 37 kj/g (9.0 kcal/g) for fat and 17 kj/g (4.0 kcal/g) for carbohydrates) was used to calculate the energy content of *Carapa procera*. Energy content (kj) = (17 × %protein) + (37 × %fat) + (17 × %carbohydrate) [22].

### Data analysis

The statistical software GenStat (12^th^ edition) was used for analysis of variance (ANOVA), and the mean values and standard deviation were calculated at 1% level of significance (p-value < 0.01).

## Results and discussion

### Phytochemicals in *Carapa procera* bark extract

The bark methanolic extract of *Carapa procera* showed the presence of abundant tannins, saponins and steroids, and less terpenoids, flavonoids and glycosides, but no alkaloids (Table 2). This result is confirmed by Seck et al. [24] who determined phytochemicals in *C*. *procera* bark extract and the presence of tannins and saponins in high amount followed by flavonoids and glycosides, but no alkaloids. However, alkaloid is present in trace amount in the bark of *Khaya senegalensis* which belongs to the same family Meliaceae [25]. Methanolic extract of *C*. *procera* seeds by Bienvenu and Marcel [26] showed the presence of alkaloids, glycosides, saponins terpenoids, steroids, tannins, and high flavonoids. Gbohaïda et al [27] also reported trace amount of alkaloids in the leaf extract of *C*. *procera*. The abundance of tannins in the bark extract of *C*. *procera* could explain its use in traditional treatment of diarrhea and may perhaps act as antitumor agents because of its ability to interact and stop different radicals produced in cells [28]. Alkaloids play an important role in drug developments, antihypertensive effects, antiarrhythmic effects, antimalarial activity, and anticancer actions [2]. Saponins have been reported by Desai et al. [29] to provides cushion for the muscles of the heart to contract properly.

**Table 2:** Qualitative test of phytochemicals in *Carapa procera* bark methanolic extract.

Parameter	*Carapa procera DC* bark extract
Alkaloids	−
Steroids	+++
Flavonoids	+
Terpenoids	++
Tannins	+++
Saponins	+++
Glycoside	+

− = Not detected, + = Trace amount, ++ = Moderate amount, +++ = Appreciable amount

### Antimicrobial activity

All the extract concentrations exhibit antimicrobial activity on the four bacterial and the fungal strains (Table 3). The 100 mg/mL extract had the highest inhibition zone on all tested organisms from 24.00 ± 0.94 to 26.67 ± 1.18, and 50 mg/mL extract showed the least inhibition zone (16.67 ± 1.24) on *Candida albicans*, while 16.00 ± 1.41, 15.33 ± 0.72, 16.00 ± 1.18, 14.67 ± 0.72 were the lowest inhibition zone for *Escherichia coli*, *Pseudomonas aeruginosa*, *Staphylococcus aureus*, and *Streptococcus pyrogen*, respectively. The outcomes revealed that the zones of inhibition observed with *C*. *procera* bark extract were lower than the antibiotics tested, but higher than DMSO. Antimicrobial activity of extracts is considered active if the inhibition zone diameter is greater than or equal to 10 mm [30]. This justifies the use of *C*. *procera* bark extract as herb. On the same microbial strains, statistical differences at P-value < 0.01 exist in their inhibition zone diameter. The antimicrobial activity of *C*. *procera* bark extract might be due to the presence of alkaloids, tannins, saponins and steroids which are known for their antimicrobial properties that act synergistically. The inhibitory activity of *C*. *procera* bark extract against both Gram-negative and Gram-positive microorganisms could be because of the presence of secondary metabolites such as alkaloids which have effect on the bacteria growth as reported by Bienvenu and Marcel [26]. Saxena et al. [2] showed flavonoids and terpenoids to exhibit antibacterial properties, as well as tannins which was abundant in the extract, described by Timothy and Idu [31] to possess antibacterial effect.

**Table 3:** Zones of Inhibition of selected microorganisms on *Carapa procera* bark methanolic extract.

Treatment (mg/mL)	Pathogens Inhibition zone diameter (mm)				
	*Candida albicans*	*Escherichia coli*	*Pseudomonas aeruginosa*	*Staphylococcus aureus*	*Streptococcus pyrogen*
100	26.33 ± 1.18^d^	25.67 ± 0.72^c^	24.00 ± 0.94^d^	24.33 ± 1.18^c^	26.67 ± 1.18^d^
50	16.67 ± 1.24^cd^	16.00 ± 1.41^bc^	15.33 ± 0.72^cd^	16.00 ± 1.18^bc^	14.67 ± 0.72^cd^
25	20.00 ± 1.24^bc^	18.33 ± 1.08^b^	17.67 ± 0.54^bc^	19.33 ± 1.18^b^	19.00 ± 0.94^bc^
12.5	23.00 ± 0.54^b^	21.00 ± 1.24^b^	20.67 ± 0.72^b^	21.33 ± 0.94^d^	22.67 ± 0.54^b^
Ciprofloxacin	0.00 ± 0.00^a^	38.00 ± 0.94^d^	39.00 ± 0.94^e^	40.00 ± 1.24^a^	38.00 ± 0.94^c^
Clotrimazole	38.67 ± 0.72^e^	0.00 ± 0.00^a^	0.00 ± 0.00^a^	0.00 ± 0.00^d^	0.00 ± 0.00^a^
DMSO	16.67 ± 0.98^b^	17.00 ± 0.94^b^	17.00 ± 0.94^bc^	15.33 ± 0.72^d^	16.00 ± 0.94^b^

Means that do not share a same letter are significantly different between rows; p-value < 0.01.

### Minimum Inhibition Concentration (MIC)

MIC determines the lowest concentration of an antimicrobial agent such as antibiotic against the growth of an organism. Low MIC value indicates that less antibiotic concentration is required to inhibit growth of the organism, and high MIC value indicates the vice versa. The MIC for the bark extract on the tested organisms is shown (Table 4). The results indicated that *S*. *aureus* was more affected by the bark methanolic extract with MIC of 3.12 mg/mL, whereas the Gram-negative bacteria varied in their sensitivity with *E*. *coli* having MIC of 25 mg/mL. The sensitivity of the Gram-positive bacteria *S*. *aureus* to the extract is confirmed in the work of Abdallah et al. [32] who reported *S*. *aureus* to be the most susceptible bacteria to all plant extracts tested among 22 bacteria. The difference in susceptibility is likely to be associated with the differences in their cell wall structure whereby Gram-positive bacteria do not have outer membrane which makes them more sensitive to substances like the extract than Gram-negative bacteria with outer membrane that act as a barrier to various substances including antibiotics [33]. The MIC for ciprofloxacin and clotrimazole (Table 4) showed *P*. *aeruginosa* to be more susceptible with MIC of 1.5625 mg/mL among the bacteria tested, whereas *C*. *albicans* had MIC of 50 mg/mL. The low MIC (3.12 mg/mL) of *C*. *procera* bark extract on *S*. *aureus* to the high MIC (6.25 mg/mL) of ciprofloxacin suggest the extract to be more effective antibacterial agent on *S*. *aureus* than all the tested bacteria. Also, the extract exhibited a high antifungal effect on *C*. *albicans* than the standard drug clotrimazole.

**Table 4:** Minimum inhibition concentration (MIC) of *Carapa procera* bark methanolic extract.

Microorganism	*Carapa procera* bark extract	Ciprofloxacin/Clotrimazole
*Candida albicans*	6.2 5 mg/mL	50 mg/mL
*Staphylococcus aureus*	3.12 mg/mL	6.25 mg/mL
*Pseudomonas aeruginosa*	12.5 mg/mL	1.56 mg/mL
*Escherichia coli*	25 mg/mL	12.5 mg/mL
*Streptococcus pyrogenes*	12.5 mg/mL	3.12 mg/mL

### Proximate composition

**Fig 1:**
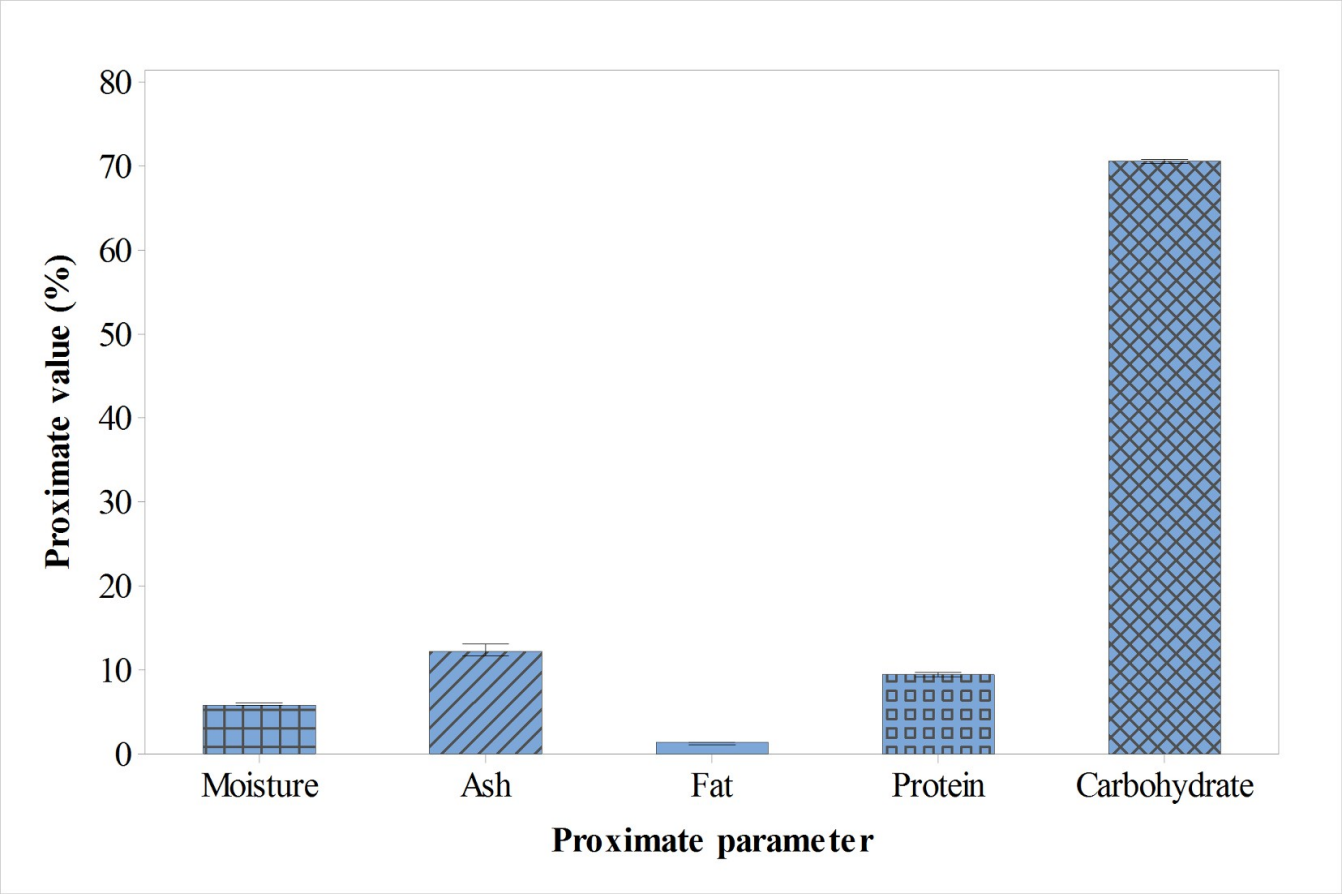
Proximate composition of dried *Carapa procera* bark.

The proximate compositions of *Carapa procera* bark are shown (Fig 1). The moisture content was found to be 6.07% which is lower than that of the seed given by Bienvenu and Marcel [26] reported to be 47.92%. The low moisture content indicates the bark is favorable for long-term storage with limit to the development of microorganisms except some molds that do grow in low moisture areas [34]. The total amount of minerals (ash) present in the bark is 12.46%, but 3.68% in the seed [26]. This indicates that even with the same plant, the amount of minerals differs in various parts with *C*. *procera* bark having higher minerals contents which could be additional benefits to its use in herbal medicine to provide good source of minerals. The crude protein contained in the bark was 9.54% which is higher than 8.13% found in *Khaya grandifoliola* which belongs to the same family of Meliaceae [35] and 8.13% found in the seed of *C*. *procera* [26]. This moderate content of crude protein available in this medicinal plant can serve as supplementary property to its application in herbal medicine. Very low amount of crude fat (1.42%) was obtained, however, 2.22% and 3.60% was recorded for the bark of *Hua gabonii* and *Scorodophleus zenkeri*, respectively [36]. The low level of crude fat in the bark of *C*. *procera* could be proposed as a weight reducing supplement because low fat meal decreases cholesterol levels [37]. *Carapa procera* bark could be considered as a good source of carbohydrate with the calculated value of 70.50%. This value is higher than what Bienvenu and Marcel [26] obtained in the seed 17.13%, but closer to 68.12% in the bark of *Khaya grandifoliola* [35]. The high total carbohydrate may serve as a good source of energy and aid digestion and assimilation of other nutrients.

The energy value of total carbohydrates, crude fat, and crude protein of *C*. *procera* bark was found to be 1413.17 kj. The high energy value obtained in the present study is mainly linked to the high carbohydrate content.

## Conclusion

The efficacy of the bark methanolic extract of *Carapa procera* shown in the result of the presence of phytochemicals and its antimicrobial activities could serve as a possible source of antibacterial for bacterial like *Escherichia coli*, *Streptococcus pyrogenes*, and antifungal against *Candida albicans* for treating different ailments. The nutritional components such as protein, minerals, and carbohydrate of *Carapa procera* bark could serve as a supplementary benefit to its application in herbal medication with an added advantage of providing basic nutrients and energy.

## Supporting information

S1 Table Dataset for zone of inhibition. (XLSX)

S2 Table Dataset for proximate composition. (XLSX)
